# Effect of demonstrator reliability and recency of last demonstration on acquisition of a socially transmitted food preference

**DOI:** 10.1098/rsos.172391

**Published:** 2018-06-20

**Authors:** Laura A. Agee, Marie-H. Monfils

**Affiliations:** University of Texas at Austin, Department of Psychology, Austin, TX, USA

**Keywords:** social learning, food choice, demonstrator reliability, conditioned taste aversion, social transmission of food preferences

## Abstract

In the social transmission of food preference paradigm, naive observer rats acquire safety information about novel food sources in the environment through social interaction with a demonstrator rat that has recently eaten said food. Research into the behavioural mechanisms governing this form of learning has found that observers show increased reliance on socially acquired information when the state of the environment makes personal examination of their surroundings risky. We aimed to (1) determine whether reliance on social information would decrease if previous reliance on social learning was unsuccessful, and (2) whether reliance on the specific demonstrator that had transmitted poor information would similarly decrease. By inducing illness in observers following consumption of a socially demonstrated food, we created an environmental situation in which reliance on socially acquired information was maladaptive. We found that under these conditions, observers showed no change in their reliance on a specific demonstrator or socially learned information in general. Our experiment also unexpectedly produced results showing that recent demonstrators were more influential in later transmissions than demonstrators that had been learned from less recently. Notably, this effect only emerged when the observer simultaneously interacted with both demonstrators, indicating that demonstrators must be in direct competition for this effect to manifest.

## 1. Introduction

For all animals, the ability to learn about and adapt to their surroundings is an essential component of survival. Gathering information on the environment is primarily done through two methods, direct experience or social learning. One of the most frequently studied forms of social learning is the social transmission of food preference (STFP), a social adaption exhibited by a number of rodent species that allows them to learn about food sources in the environment through interaction with conspecifics. In the standard laboratory model of STFP, an observer rat is allowed to interact with a demonstrator rat that has recently eaten from a novel food source. Following this interaction, the observer is isolated and given simultaneous access to the demonstrated food and a second novel, undemonstrated food. Observers reliably display a preference for the demonstrated food, indicating that social learning has occurred [1–3]. STFPs are pheromone mediated, with transmission depending on the concurrent olfactory reception of odour cues associated with the demonstrated food and carbon disulfide (CS_2_) present on the breath of the demonstrator [4–5], and long-lasting [6], with behavioural signs of memory decay not manifesting until months following acquisition [7]. Due partially to its reliability and ease of use, the STFP model has been used by a host of studies to test the effect of various environmental and social factors on the degree to which an animal relies on information acquired via social learning (for review, see Galef [8]).

Acquiring information through learning without relying on social cues/interactions (asocial learning) requires that an animal spend time and energy investigating its surroundings and risk exposing itself to danger. Learning about the environment through social observation allows the learner to avoid this energy expenditure and risk. Conversely, the learner can only obtain what information it can intuit through social cues, meaning that information obtained via social learning is inevitably limited and has the potential to be outdated or inaccurate. Theoretical models posit that due to this cost/reliability trade-off between individual learning and social learning, demonstrator, environmental and individual characteristics that alter the risk of asocial learning or the reliability of social learning should affect the degree to which an animal relies on either strategy (e.g. an unstable environment would increase the chance that socially acquired information would be outdated; therefore, social learning should be relied on less) (for review, see Rendell *et al*., [9]). A number of predictions about the effect that the characteristics of the observer/environment might have on reliance on social versus asocial learning have been experimentally confirmed in the STFP model [10–13], possibly implying that information acquired via STFP may be encoded in such a way that it is specifically ‘tagged’ as being socially acquired.

A number of studies have confirmed that some of the demonstrator characteristics that are predicted to be influential such as kinship/familiarity [14–16] or dominance status [16,17] influence the strength of socially learned fear behaviours in rodents as predicted. While these findings confirm that demonstrator characteristics do have the potential to influence reliance on some types of social learning, similar studies using STFP as their model of social learning have repeatedly produced negative results [12,18]. The most surprising of these negative results have been the numerous studies demonstrating that the health status of a demonstrator at the time of interaction has no effect on the strength of a STFP, e.g. an ill demonstrator is just as effective as a healthy demonstrator in transmitting a food preference to its observer [19–21]. Notably, the success or failure of all these studies relies on the assumption that the observer is aware at the time of interaction that it is receiving information from its demonstrator. As acquisition of an STFP is pheromone mediated [4] and in no way requires that the observer attend to or interpret the behaviour of its demonstrator, it is entirely possible that the transfer of information occurs without the observer's conscious awareness. This idea is further supported by past research which has found that not only is the presence of a conspecific in no way necessary for an STFP to be acquired as long as the observer is exposed to CS2 in combination with a novel scent [1,4], but that observers are capable of acquiring an STFP of equal strength to that acquired by awake controls while under anaesthesia [22].

While observers might not be aware that social learning is occurring during acquisition of an STFP, the aforementioned findings in regards to the effects of environment and observer characteristics certainly suggest that STFPs are relied on as social information. We posited that if this were the case, we might be able to alter the tendency for rats to rely on socially acquired information by intervening after the STFP had been encoded. We also posited that this might be sufficient to alter the response of observers to a specific demonstrator. We designed a set of experiments aimed at determining (1) whether observers would rely less on socially acquired information after past reliance had resulted in illness and (2) whether observers would selectively rely less on information obtained from the specific individual that had supplied the unreliable information.

## 2. Experiment 1: social transmission of food preference verification

### 2.1. Overview

The purpose of Experiment 1 was to verify the base STFP behavioural paradigm in our laboratory. In addition, to optimize the ability of subsequent experiments to pick up on small changes in preference, we tested both high and low interaction times between observers and demonstrators.

### 2.2. Material and methods

#### 2.2.1. Subjects

Subjects were 32 male Sprague-Dawley rats weighing 250–300 g and obtained from Harlan, now known as Envigo (Houston, TX, USA). All rats used for this experiment had been run through unrelated fear conditioning experiments prior to the start of Experiment 1.

#### 2.2.2. Diets

We prepared two novel diets, diet cinnamon (Cin) and diet cocoa (Co), by mixing a 100 g of powdered 5LL2 Purina rodent chow with either 1 g of McCormick ground cinnamon (diet Cin) or 2 g Hershey cocoa powder (diet Co).

#### 2.2.3. Apparatus

All phases of the experiment took place in standard rat cages (10.5′′ × 19′′ × 8′′) with the exception of the interaction phase which took place in a large plastic bin (19.875′′ × 15.5′′ × 14.75′′) with woodchip bedding covering the bottom. Bedding was refreshed between groups. Novel foods were presented to rats in small circular bowls secured to the floor of the cage using adhesive strips.

#### 2.2.4. Procedure

Rats arrived in triads but were rehoused in pairs at least 10 days before the start of the experiment so that cage-mates would have time to become socially well-acquainted. All rats received distinct tail markings with sharpie so that they could be distinguished from their cage-mate. One rat in each pair was randomly assigned to the ‘Observer' condition while their cage-mate was assigned to the ‘Demonstrator' condition. Three cohorts of animals were run through the procedure, with observers in two cohorts receiving low social interaction (15 min duration) with their demonstrator (*n* = 10) and one cohort receiving high social interaction (30 min duration) with their demonstrator (*n* = 6). Prior to the start of the experiment all rats were allowed ad libitum access to pelleted 5LL2 Purina rodent chow. At the end of social familiarization, demonstrator rats were moved to single housing and food deprived for 26–28 h while observers remained in the home cage with ad libitum food access.

— *Step 1*. Following food deprivation, demonstrators were moved to a room adjacent to the colony and given access to 25 g of either diet Co or diet Cin for 30 min. The food bowl was removed from the cage and weighed at the end of the feeding period to ensure that all demonstrators had eaten a sufficient amount of the novel flavour.
— *Step 2.* Demonstrators were returned to the colony and placed in a large plastic bin with their observer where the two were allowed to freely interact with each other for either 15 min (low social interaction condition) or 30 min (high social interaction condition).
— *Step 3.* Once the demonstration period had passed, observers were immediately moved to a standard rat cage containing two pre-weighed bowls, one of which contained 25 g of diet Co and one of which contained 25 g of diet Cin. After 18 h had elapsed, observers were returned to their home cage and the remaining food in each bowl was weighed to determine the amount of each diet which the observer had eaten. Notably, observers were not food deprived before their choice test because (1) the 18 h length of the choice test made this an unnecessary stressor and (2) we hoped that removing any immediate strong motivation produce by hunger would encourage observers to be more ‘picky' in choosing which food to consume, thus increasing the sensitivity of our test to small differences in flavour preference. Subsequent experiments use this strategy for the same reasons.

### 2.3. Results

Where it is statistically feasible to do so, the dependent variable used for all statistical tests in Experiments 1–3 is the percent preference for the demonstrated diet (following STFP) or the percent preference for the LiCl paired flavour (following CTA). Percent preference for a given food type is calculated using the following formulas: (Diet 1: D_1_/(D_1_+D_2_) × 100; Diet 2: D_2_/(D_1_+D_2_) × 100) where *D*_n_ = the total grams eaten of diet n at the choice test. Significance levels for all choice test data were determined using a one-sample *t*-test tested against a *μ* = 50% except where otherwise noted.

#### 2.3.1. STFP choice test results

**Figure 1.**
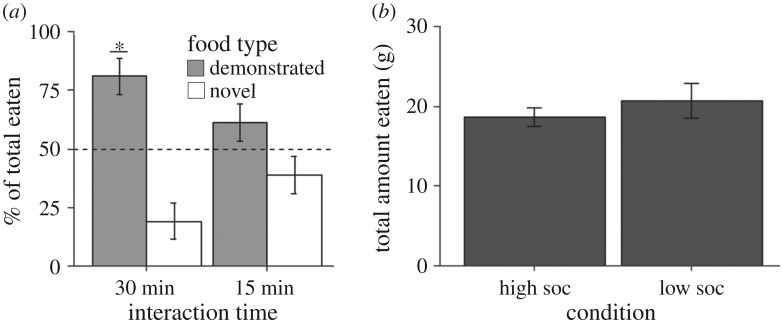
Observers display a preference for a demonstrated novel flavour over an undemonstrated novel flavour. (*a*) Mean (±s.e.m) percent preference for a demonstrated flavour versus a novel flavour at a choice test following either a 15- or 30-min interaction with their demonstrator. Observers in the high interaction time (30 min) but not the low interaction time (15 min) condition displayed a significant preference for the demonstrated flavour. (*b*) The mean (±s.e.m.) total amount in grams eaten by observers during the choice test as a function of interaction time. Results indicate no effect of interaction time on food consumption (*p < 0.05).

Figure 1*a* visualizes the percent preference of observers in each group for the demonstrated and novel food. We confirmed that observers in both interaction conditions ate a similar amount to one another (high interaction: 18.62 g ± 1.178 g; low interaction: 20.71 g ± 2.21 g, figure 1*b*), and found that there was only one case of an observer consuming the entire 25 g of a given flavour in the present experiment, making a ceiling effect unlikely. The effects of group and flavour on the percent total eaten of the demonstrated flavour were analysed using a two-way ANOVA. Interaction time (*F*_1,12_ = 3.037, *p* > 0.1) and flavour (*F*_1,12_ = 3.489, *p* = 0.086) did not reach significance, and no interaction between the two was detected (*F*_1,12 _= 0.316, *p* > 0.1), verifying our use of the *μ* = 50% marker for subsequent *t*-tests. Observers in the 30-min interaction time displayed a significant preference for the demonstrated flavour (*t*_5_ = 4.01, *p* = 0.01), while the strength of preference for the demonstrated flavour in the 15-min interaction time group did not reach significance (*t*_9_ = 1.43, *p* > 0.1). Notably, seven of the 10 observers in the low social interaction group did display a preference greater than 60% for the demonstrated flavour.

## 3. Experiment 2: effect of demonstrator reliability—within-subjects design

### 3.1. Overview

This experiment aimed to test whether observers were able to identify and selectively discriminate against information obtained from a demonstrator that had previously provided unreliable information. Observers (1) received demonstrations from two different rats using the standard STFP protocol described in Experiment 1, (2) were allowed access to each of the demonstrated foods and (3) were made ill via injection of a lithium chloride (LiCl) solution, a strong emetic, following the consumption of one of these foods but not the other. We then had both demonstrator rats eat two entirely novel flavours and allowed them to interact simultaneously with their observer. Following this final demonstration, the observers were given limited access to both of the demonstrated flavours.

### 3.2. Material and methods

#### 3.2.1. Subjects

Subjects for this experiment were 72 male Sprague-Dawley rats obtained from Harlan and weighing between 275 and 350 g. Two cohorts (*n* = 36) had been fear conditioned prior to the start of the experiment. All rats remained in their triads for at least 2 weeks before the experiment proper began.

#### 3.2.2. Diets

Four novel diets were used for this experiment, including diets Cin and Co which were formulated as described in Experiment 1. In addition, two new diets were formulated by mixing 100 g of 5LL2 powdered rodent chow with either 2.4 g powdered marjoram (diet Mar) or 1 g ground anise (diet Ani).

#### 3.2.3. Apparatus

During food deprivation, demonstrators were separated from their cage-mates by an aluminium barrier installed at the midsection of their home cage and secured by hooks inserted into each corner. A number of holes large enough for the rats to insert their snout through were drilled into the barrier, allowing limited social interaction during the deprivation period. Novel diets were presented in hanging food cups constructed from 12-gauge steel utility wire and 4 oz. glass jars. Small bowls were secured to the bottom of the cage just below the hanging food cups to catch any spillage. All interaction sessions took place in the interaction bin described in the methods section of Experiment 1.

#### 3.2.4. Procedure

**Figure 2.**
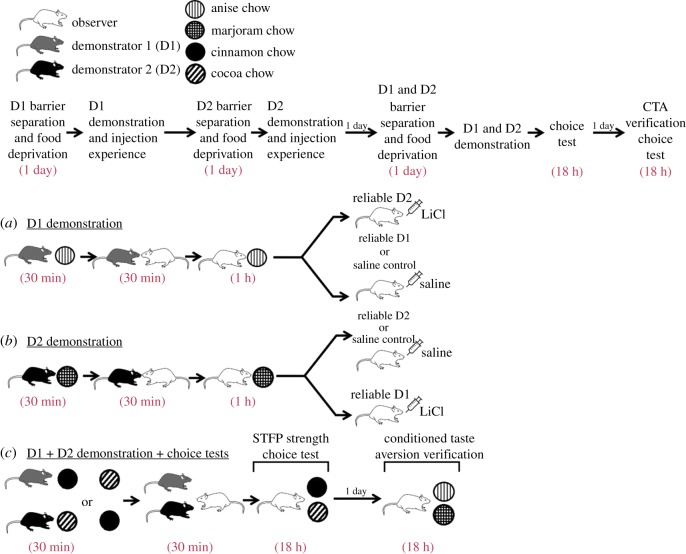
Experiment 2 behavioural procedure. The top flowchart lists housing details during the experiment and gives an abbreviated outline of the order of procedures. The graphics below display the specifics of the each procedure. In brief, observers acquired two STFPs through interaction with (*a*) demonstrator 1 (D1) followed by (*b*) interaction with demonstrator 2 (D2) 2 days later. After each interaction, observers were given access to the demonstrated flavour and then injected with either LiCl to induce illness and a conditioned taste aversion or Saline as a control. Rats that had demonstrated a flavour that was followed up with a LiCl injection were tagged as unreliable. (*c*) Two days following the second demonstration, observers were allowed to learn two new STFPs via simultaneous interaction with their demonstrators and then completed a choice test to determine whether they displayed increased reliance on information obtained from the reliable demonstrator. Following the end of the main experiment, observers were given a second choice test between the flavours which had been followed up with an injection to ensure that they had acquired a conditioned taste aversion to the LiCl paired flavour.

For reference, the full behavioural procedure for Experiment 2 is graphically represented in figure 2. Prior to the start of the experiment, the tails of each rat was marked with a sharpie so that they would be distinguishable from their cage-mates. Each rat in a triad was then randomly assigned to one of the following conditions: Observer, Demonstrator 1 (D1), or Demonstrator 2 (D2) (*n* = 24 for each condition). Observers were then assigned to the Saline (Sal) Control (Sal/Sal injection order; *n* = 6), Reliable D1 (Sal/LiCl injection order; *n* = 9) or Reliable D2 (LiCl/Sal injection order; *n* = 9) condition. Notably, while the Reliable D1 (Sal/LiCl) and Reliable D2 (LiCl/Sal) assigned observers were present in the first three cohorts that we ran, the Saline Control (Sal/Sal) assigned observers were all in the fourth and final cohort. The experiment proceeded as follows:

— *Demonstration 1.* (Note: For the following procedures food weights were taken before and after every consumption period to ensure sufficient consumption of the novel flavour by demonstrator/observer rats. This will remain the case for all subsequent procedures and experiments.) Rats assigned to the D1 condition were barrier separated from their cage-mates and food deprived over a 24 h period. Following food deprivation, D1 rats were moved to a room adjacent to the colony and allowed 30 min of access to diet Ani. D1 rats were then returned to the colony room and placed in the interaction bin with the Obs assigned rat in their triad for 30 min while D2 remained in the home cage. Following the interaction period, D1 rats were placed in single housing for 24 h to ensure that the scent of diet Ani was no longer on their breath before being returned to their home cage. Observers were moved to an adjacent room and given 1 h of access to diet Ani, after which they were injected intraperitoneally (i.p.) with 20 ml kg^−1^ of saline if assigned to the Reliable D1 (Sal/LiCl) or Saline control (Sal/Sal) conditions or 0.15 M LiCl dissolved in saline if assigned to the Reliable D2 (LiCl/Sal) condition. Injections of LiCl at this concentration and volume are known to result in gastrointestinal distress severe enough to induce display a clear aversion towards diets eaten prior to injection [23]. Observers were then returned to their home cage to recover. All LiCl-injected observers were kept under observation until the acute symptoms of gastrointestinal distress has passed.
— *Demonstration 2.* Twenty-four hours following the end of Demonstration 1, D2 rats were barrier separated and food deprived for 24 h. After food deprivation was complete, D2 rats were transported to a room adjacent to the colony and allowed 30 min of access to diet Mar. From here, the procedure proceeded identically to Demonstration 1 except that Observers in the Reliable D2 (LiCl/Sal) condition were injected i.p. with saline, while rats in the Reliable D1 (Sal/LiCl) condition received a LiCl injection. Saline Controls (Sal/Sal) once again received a saline injection.
— *Final demonstration/STFP choice test.* Twenty-four hours following Demonstration 2, D1 and D2 rats were both barrier separated from their Observer assigned cage-mate and food deprived for 24 h. At the end of this food deprivation experience, D1 and D2 rats were moved to an adjacent room and given 30 min of access to either diet Cin or diet Co. Whether diet Cin or Co was eaten by D1 or D2 was counterbalanced to control for possible effects of demonstrated flavour. Following this consumption period, D1 and D2 were returned to the colony room and placed in the interaction bin to interact simultaneously with the Observer. This interaction period was recorded by an overhead camera and was later scored offline for various social behaviours of the Observer direct towards a given demonstrator. After the interaction period was over, the Observer was moved to a standard rat cage in which two hanging feeders were present, one containing 30 g of diet Co and one containing 30 g of diet Cin. Observers were allowed 18 h access to both flavours, after which they were returned to their home cage with D1 and D2 and the total amount of each diet that was eaten was weighed to determine which demonstrator's information the Observer had relied on more.
— *CTA Verification.* Twenty-four hours following the end of the STFP choice test, Observers were moved to a standard rat cage containing two hanging jars, one of which contained 30 g of diet Ani and one of which contained 30 g of diet Mar. Observers were allowed 18 h of access to these food cups, after which the remaining amount of each diet was weighed. This provided solid behavioural evidence that observers had developed a conditioned taste aversion against the diet after which they had been injected with LiCl.

### 3.3. Results

#### 3.3.1. STFP choice test results

**Figure 3.**
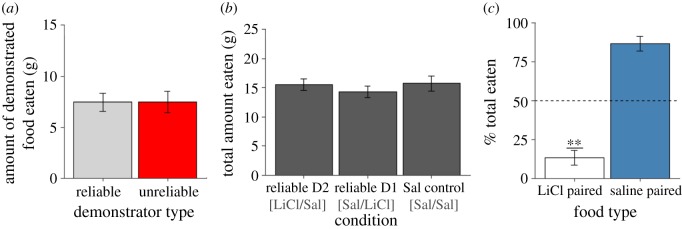
Demonstrator reliability does not affect subsequent learning. (*a*) The mean (±s.e.m.) amount in grams consumed of diets demonstrated by unreliable demonstrators (red bar) versus reliable demonstrators (grey bar) in Experiment 2. Rats that had experienced an aversive outcome after consuming a food they had acquired an STFP for from the Unreliable demonstrator showed no change in their tendency to rely on new information obtained from that rat. (*b*) Experimental condition did not appear to affect the mean (±s.e.m.) total amount in grams eaten by observer rats at the STFP strength choice test. (*c*) The average preference of observers (±s.e.m.) for the demonstrated flavour that had later been followed up by a LiCl injection (white bar) as compared to the demonstrated flavour that had been followed up with a saline injection (blue bar) as assessed during the conditioned taste aversion verification choice test in Experiment 2. Results indicate that observers successfully acquired the taste aversion. (**p < 0.01).

**Figure 4.**
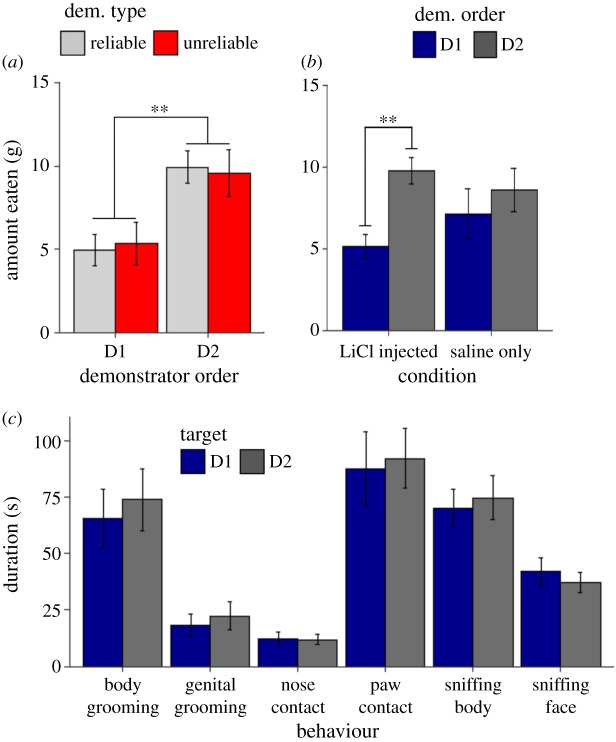
Relative recency of past STFP acquisition from a given demonstrator (i.e. demonstrator order) facilitates subsequent learning from that demonstrator. The average amount in grams (±s.e.m.) eaten of the demonstrated diets separated by (*a*) demonstrator reliability and order for observers in the LiCl-injected groups or (*b*) demonstrator order and injection condition. (*c*) The average duration in seconds (±s.e.m.) spent by observers socially attending to each demonstrator. We found no significant differences in observer-initiated social contact between the two demonstrators for any of the scored behaviours (**p < 0.01).

In order to identify effects of demonstrator order, all statistical tests run on data from the choice test following the final interaction phase of the experiment uses the total grams of the demonstrated food eaten as the dependent variable. In order to ensure that this measure would not be confounded by differences in overall food consumption, the average (±s.e.m.) total amount eaten in grams during the STFP choice test for each condition was calculated (Reliable D1 [Sal/LiCl]: 14.311 g ± 0.965 g; Reliable D2 [LiCl/Sal]: 15.533 g ± 1.017 g; Saline Control [Sal/Sal]: 15.733 g ± 1.298 g, figure 3*b*), and a one-way ANOVA confirmed that there was not a significant difference in the total amount eaten by observers in any of the three conditions (*F*_2,21_ = 0.529, *p* > 0.1). The difference in the grams eaten of the demonstrated flavour for observers in all conditions was analysed using a mixed-design ANOVA with a within-subjects variable of demonstrator order (i.e. the relative time because a given demonstrator had been learned from) and a between-subjects variable of condition. The ANOVA indicated that while condition had no significant effect on the amount of the demonstrated food eaten by the observer in the choice test (*F*_2,21_ = 0.53, *p* > 0.1, figure 3*a*), the order of the demonstrator had a significant effect on how much of the demonstrated flavour was consumed (*F*_1,21_ = 7.55, *p* = 0.012, figure 4*a*). The ANOVA also found no interaction between demonstrator order and condition, indicating a lack of effect of demonstrator reliability (*F*_2,21_ = 0.695, *p* > 0.1, figure 4*a*). As follow up *t*-tests were all between subjects, the percent preference for the demonstrated food was used as the dependent variable in order to directly control for possible effects of differences in total consumption. Data for observers that had received LiCl injections (Reliable D2 [LiCl/Sal] and Reliable D1 [Sal/LiCl] conditions) and data from observers that had only received saline injections (Saline Control [Sal/Sal] condition) were separated and a paired *t*-test was run on each to determine whether demonstrator order retained an effect based on injection condition. Only observers that had received a LiCl injection were found to display the effect of demonstrator order when tested in this way (saline only: *t*_5_ = −0.566, *p* > 0.1; LiCl injected: *t*_17_ = 3.1, *p* < 0.01, figure 4*b*). These *t*-tests were run based on the *a priori* (i.e. before running the Saline Control group) prediction formed after our detection of the demonstrator order in our LiCl receiving observers that the LiCl injections might be driving the demonstrator order effect. It should be noted that an independent *t*-test comparing the percent preference for the D2 demonstrated flavour between the LiCl-injected and saline only observers did not turn up a significant difference (*t*_24_ = −1.08, *p* > 0.1). In order to help gauge the likelihood that the blunted effect of demonstrator order seen in our Saline Control (Sal/Sal) group would persist with a larger sample size, we calculated Cohen's *d*_z_ as a measure of effect size for demonstrator order and found that our sample data provided an effect size of 0.231, generally considered to be a small effect. A power analysis completed using the G*Power 3.1 software [24] found that with an effect size of 0.231, 149 subjects in total would have to be run in order to verify whether the observed effect was real. For LiCl-injected observers, Cohen's *d*_z_ was calculated to be 0.782, close to what is considered a large effect.

#### 3.3.2. CTA choice test results

The results of the choice test that was run at the end of the experiment verified that the conditioned taste aversions had developed as expected in the observers that had received LiCl injections (*t* = −7.57, *p* < 0.001, figure 3*c*).

#### 3.3.3. Social behaviour

Observers were scored based on the total duration of a number of social behaviours initiated by them towards a given demonstrator. The following behaviours were scored: sniffing near face, sniffing near back, nose contact, paw contact, body grooming and genital grooming. All behaviours were scored from video taken during the interaction period using the Behavioral Observation Research Interactive Software (BORIS) [25]. The duration in seconds of individual behaviours during social interaction in all groups was analysed using a two-way mixed-design ANOVA with demonstrator order as a within-subject factor and condition as the between-subjects factor. The dependent variable was Box-Cox transformed for the genital grooming, face sniffing, nose contact and paw contact behaviours in order to bring the data in line with the assumptions of the ANOVA. The ANOVA picked up no significant effects of demonstrator order (figure 4*c*), condition or interaction between the two on the average duration of the scored behaviours (table 1).

**Table 1.** Statistical analyses of the duration in seconds of different social behaviours based on demonstrator order. All findings indicate a *p* > 0.05.

	ANOVA results		
behaviour	condition	demonstrator order	condition × demonstrator order
body grooming	*F*_2,21 _= 0.703	*F*_1,21_ = 0.001	*F*_2,21_ = 1.975
genital grooming	*F*_2,21 _= 0.4225	*F*_1,21_ = 0.3810	*F*_2,21_ = 0.4421
nose contact	*F*_2,21 _= 0.5123	*F*_1,21_ = 0.0302	*F*_2,21_ = 0.0264
paw contact	*F*_2,21 _= 2.2	*F*_1,21_ = 0.0255	*F*_2,21_ = 0.689
sniffing body	*F*_2,21 _= 1.92	*F*_1,21_ = 1.138	*F*_2,21_ = 1.166
sniffing face	*F*_2,21 _= 2.1830	*F*_1,21_ = 0.767	*F*_2,21_ = 1.1863

## 4. Experiment 3: effect of demonstrator reliability—between-subjects design

### 4.1. Overview

Experiment 3 was designed to confirm that the null results in regards to demonstrator reliability that were observed following Experiment 2 were not due to difficulty on the part of observer in identifying whether food information was coming from demonstrator 1 or demonstrator 2. In addition, we wished to determine whether overall reliance on social information was decreased following illness resulting from past reliance on social information. As in Experiment 2 observers were given access to a food they had recently acquired a STFP for and then injected with LiCl or Saline, which would serve as their only injection experience. A second demonstration of a novel flavour was completed by either the same or a novel demonstrator. This design allowed us to separate out possible effects of having learned from the demonstrator recently, effects of an aversive social learning experience, and the effects of demonstrator reliability. We hypothesized that observers would learn better from a demonstrator that they had already acquired an STFP from than a novel demonstrator, regardless of the quality of the information that had been transmitted by the familiar demonstrator. In addition, we hypothesized that observers would rely less on social information in general if previous reliance had resulted in an aversive event (i.e. a LiCl injection).

### 4.2. Material and methods

#### 4.2.1. Subjects

Subjects were 36 male Sprague-Dawley rats obtained from Harlan and weighing between 275 and 300 g*.* Rats arrived and were housed in triads.

#### 4.2.2. Diets

Diets Cin, Co, Mar and Ani were formulated as described in Experiments 1 and 2.

#### 4.2.3. Apparatus

All apparatuses used for this experiment are described in the methods section of Experiment 2.

#### 4.2.4. Procedure

**Figure 5.**
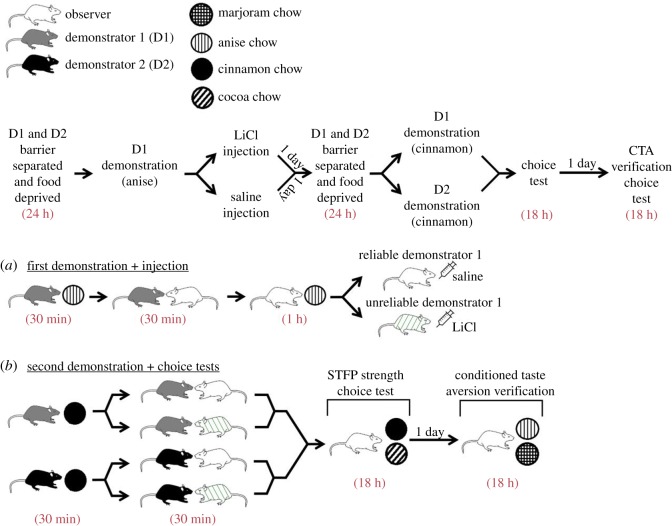
Experiment 3 behavioural procedure. The top flowchart lists housing details during the experiment and gives an abbreviated outline of the order of procedures. The graphics below display the specifics of each procedure. (*a*) Observers interact with a demonstrator that has recently eaten anise-flavoured chow. Afterwards, they are given 1 h to eat anise chow and then injected with either LiCl to induce gastrointestinal distress or saline as a control. (*b*) Two days following this first demonstration, observers interact with either the demonstrator they learned from earlier OR a novel demonstrator after they have eaten cinnamon flavoured chow. Afterwards, observers are given a choice test between cinnamon chow and the novel cocoa flavoured chow. Following the end of the main experiment, observers were given a second choice test between anise chow and the novel flavoured marjoram chow to verify that LiCl-injected observers had acquired a conditioned taste aversion.

For reference, the full behavioural procedure for Experiment 3 is graphically represented in figure 5. As in Experiment 2, each rat in a triad was assigned to the Observer, D1 or D2 condition. Cages were randomly assigned to the D1 or D2 final demonstrator condition and were then further subdivided into the Reliable D1 and Unreliable D1 conditions. This resulted in a total of four possible conditions for observers: (1) D1 final demonstrator, reliable D1; (2) D1 final demonstrator, unreliable D1; (3) D2 final demonstrator, reliable D1; (4) D2 final demonstrator, unreliable D1 (*n* = 3 for each condition). The experiment ran as follows:

— *Injection and first demonstration.* Both D1 and D2 were barrier separated from their observer cage-mate and food deprived for 24 h. Though only D1 assigned rats were demonstrating at this stage, the D2 barrier separation and food deprivation was necessary to keep the interaction protocol consistent between the first and second demonstration phase for all rats. Following food deprivation, D1 rats were moved to an adjacent room and allowed access to diet Ani for 30 min while D2 rats were single housed and given ad libitum food access. Following this 30 min consumption period, D1 rats were returned to the colony and placed in the interaction bin with their observer for 30 min. Following the interaction period, D1 was moved to the same cage as D2 for 24 h to allow for the scent of diet Ani to fade. Observers were given 1 h of access to diet Ani in a hanging food cup, after which they received a 20 ml kg^−1^ injection of saline if assigned to the Reliable D1 condition or a 20 ml kg^−1^ injection of 0.15 M LiCl if assigned to the Unreliable D1 condition. Following their injection, observers were returned to their home cage to recover. As in Experiment 2, LiCl-injected rats were closely monitored to ensure that they did not display any signs of pain or stress beyond what would be expected.
— *Second demonstration and choice test.* Twenty-four hours following the end of the previous phase of the experiment, D1 and D2 were returned to the home cage, barrier separated and food deprived for 24 h. Following this food deprivation period, in cages in which observer were assigned to the D1 final demonstrator condition, D1 was moved to an adjacent room and allowed 30 min of access to diet Cin before being returned to the colony and allowed to interact with their observer for 30 min. For observers assigned to the D2 final demonstrator condition, D2 was put though this same protocol. Following their interaction with either D1 or D2, observers were moved to a cage outfitted with two hanging food cups, one containing the demonstrated flavour (diet Cin) and one containing a novel undemonstrated flavour (diet Co). Observers were allowed 18 h of access to 30 g of each of these diets before being returned to their home cage. The remaining amount of food present in each bowl as weighed out and the total amount eaten of each flavour by the observer was calculated.
— *CTA verification.* Twenty-four hours following the end of the choice test between diets Cin and Co, observers were once again moved to a rat cage containing two hanging food cups one containing 30 g of the flavour eaten before the first demonstration, diet Ani and the other containing 30 g of the novel and undemonstrated diet Mar. Observers were again given 18 h of access to the food cups, after which the remaining food was weighed and the total amount of each flavour eaten was calculated.

### 4.3. Results

#### 4.3.1. STFP choice test results

**Figure 6.**
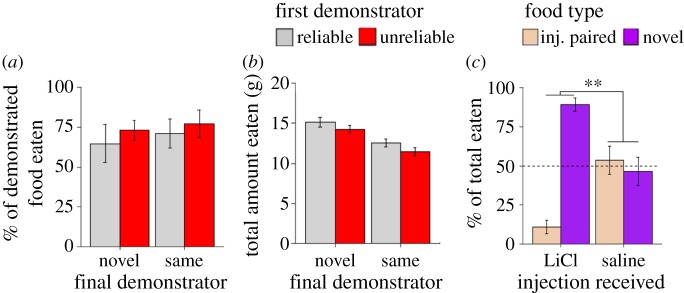
Demonstrator novelty and past reliability do not affect subsequent learning in a between-subjects model. (*a*) The mean (±s.e.m.) preference of observers for the second flavour they received a demonstration for in Experiment 3 based on the novelty of the demonstrator and the injection they received following the first demonstration. (*b*) The mean total (±s.e.m.) amount in grams eaten by observers during the STFP strength choice test in Experiment 3. (*c*) Results of the conditioned taste aversion verification choice test on the final day. Observers made ill by LiCl injection following consumption of the demonstrated diet showed a significant aversion towards that diet (**p < 0.01).

The percent consumption of cinnamon flavoured chow following observer's second interaction was analysed using a two-way between-subjects ANOVA with demonstrator novelty and injection type as a between-subjects factors. No significant effect of demonstrator novelty (*F*_1,8 _= 0.329, *p* > 0.1), injection type (*F*_1,8 _= 0.613, *p* > 0.1) and no interaction between the two (*F*_1,8_ = 0.017, *p* > 0.1) (figure 6*a*) was detected. Given our small sample size per cell (*n* = 3), a post hoc power analysis was completed to determine whether running additional animals to attempt to increase the power of our study was reasonable. Effect sizes were calculated for the main effects of demonstrator novelty (*η*^2^ = 0.0266) and injection type (*η*^2^ = 0.0486), as well as the interaction between the two (*η*^2 ^= 0.0014), which according to Miles & Shevlin's [26] general guidelines, all fall short of even a medium effect size (*η*^2^ = 0.06). A post hoc power analyses was completed using the G*Power 3.1 software and we found that a sample size of 150 would be required to achieve even 0.8 power with the largest effect obtained from this experiment. As small samples tend to overestimate effects size, that our calculated effects are as small as they are suggests that either there is no effect or that any effects we would uncover through running additional subjects would be too small to have much practical significance.

#### 4.3.2. Total eaten during STFP choice test

Since visualization of the total grams consumed across conditions produced an interesting trend (figure 6*b*), as a post hoc analysis, a second two-way between-subjects ANOVA with demonstrator novelty and injection type as between-subjects factors was run on the total amount eaten during the STFP verification choice test in Experiment 3. As with the percent consumption, no significant effect of demonstrator novelty (*F*_1,8 _= 3.42, *p* > 0.1), injection type (*F*_1,8 _= 0.4482, *p* > 0.1) or any interaction between the two (*F*_1,8 _= 0.0104, *p* > 0.1). As with the last analysis, effect sizes were calculated for the main effect of demonstrator novelty (*η*^2 ^= 0.2218) and injection type (*η*^2 ^= 0.036) and for the interaction between the two (*η*^2 ^= 0.0014). A post hoc power analysis indicates that a sample size of around 30 subjects would be sufficient to reach a power level of 0.8 for the main effect of demonstrator order on total amount eaten. We should thus exercise caution in interpreting a lack of effect of demonstrator novelty in this instance, because the effect size estimated was reasonably large.

#### 4.3.3. CTA choice test results

As all observers had been injected following consumption of the same flavour, the development of a CTA was assessed using an independent *t*-test comparing the percent total eaten of the flavour in the LiCl injected to the saline-injected groups. As expected, LiCl-injected rats displayed a significantly lower preference for anise-flavoured chow over marjoram flavoured chow (*t*_24_ = −4.23, *p* < 0.01, figure 6*c*). It is worth noting that a one-way *t*-test (*t*_5_ = 0.391, *p* > 0.1) found that observers in the saline-injected group did not display a significant preference for anise-flavoured chow over marjoram flavoured chow. This is unusual given that they had interacted with a demonstrator that had eaten anise-flavoured chow just a few days prior.

## 5. Discussion

Overall, our findings indicate that observers do not alter their tendency to learn from a specific demonstrator based of the quality of information transmitted in the past. Furthermore, observers did not display any decrease in reliance on socially transmitted food preferences as a whole, even when past reliance resulted in illness. While the former result is unsurprising given the findings of previous research, which suggest that observers do not take into account demonstrator characteristics when choosing whether to rely on a STFP, the latter is unexpected as rats have been shown to alter their reliability on STFPs based on the degree to which the environment supports individual learning over social learning. As reliance on socially acquired information is only adaptive as far as it allows an animal to circumvent danger, an experience contradicting the assumed increased safety of relying on social information would be expected to promote individual learning.

This study is not the first to demonstrate that rats are not as sensitive to clear threats in their environment that should alter their tendency to rely on a socially transmitted food preference. Experiments conducted by Galef & Whiskin [27] and Galef & Yarkovsky [28], which test the effects of exposing observer to clear evidence of danger at the food site (e.g. the presence a predator's scent), also produced null results. Our findings may simply be another example of this counterintuitive lack of observer sensitivity to explicit environmental threats. This said, an isolated aversive event resulting from reliance on social learning may simply not have been sufficient to induce a decrease in reliance on social information. It is quite possible that if observers were to go through multiple trials of learning an STFP, eating the demonstrated food and being injected with LiCl, we might observe the predicted effect. It should also be stated that in interpreting the results of Experiment 3 it is important to take into account the small sample size per cell (*n* = 3). The effect sizes calculated from our samples were also very small, so we believe that it is unlikely that our design would have yielded an effect even with a reasonably larger sample size (a power of 0.8 estimates sample sizes of over 100). Still, the possibility remains that a main effect of social information reliability does exist and our sample size was simply insufficient to detect it. This interpretation is further complicated by the fact that, due to our use of only one of the two choice test diets, we cannot statistically validate that the rats used in Experiment 3 obtained a socially transmitted food preference. Follow-up research using larger sample sizes, varying LiCl injection trials and counterbalanced diet presentations will be required to affirm that our findings from this experiment are valid.

Unexpectedly, the most interesting results of our experiments came from the order in which demonstrators were initially learned from in Experiment 2. When observers were given simultaneous demonstrations of two distinct novel flavours eaten by two rats they had learned from in the past, observers subsequently exhibited a strong preference for the novel flavour demonstrated by the rat that they had most recently learned from. As analysis of observers' social behaviour during this simultaneous demonstration showed no difference in the observers' treatment of D1 and D2, it seems likely that the effect of demonstrator order detected in this model may unveil information about the way that information is processed during acquisition of a STFP. Whether this is an effect of the order of demonstrations or the time elapsed since the initial demonstration is not clear at this point. The possibility that the subsequent enhanced preference displayed by the observer for D2's diet was somehow related to the flavour demonstrated during the first interaction should also be acknowledged. As the flavour demonstrated during the initial interactions in Experiment 2 was always the same based on the demonstrator's order of presentation, demonstrator 1 always ate anise-flavoured chow and demonstrator 2 always ate marjoram flavoured chow. However, this explanation would only make adaptive sense in a context in which observers greatly favour marjoram chow and are able to link the identity of D2 to the demonstration of this favoured flavour. As the findings of this very study strongly indicate that observers would not be capable of doing this, this explanation seems unlikely.

Surprisingly, observers in Experiment 3 did not display a significant increase in their preference for the second demonstrated flavour over a novel undemonstrated flavour if they had learned from their demonstrator in the past, though notably our results did trend non-significantly in the direction of there being a demonstrator effect. There are two likely explanations for this: (1) the decreased sensitivity of our between-subjects design for detecting small changes in observer preferences washed out the effect of previous learning experiences with the demonstrator or (2) this effect only manifests when observers are receiving olfactory inputs from multiple demonstrators simultaneously. The latter explanation would suggest that there is something unique about the way social information is processed when multiple demonstrators are present. While previous work using a model of STFP in which multiple demonstrators are presented simultaneously is limited, there are some articles that suggest this might be the case. For example, when interacting with multiple demonstrators, some that have eaten cinnamon flavoured chow and some that have eaten cocoa flavoured chow, observer rats do display a preference for the flavour that is demonstrated by the majority of their conspecifics [29]. This might indicate that observers are processing olfactory information distinguishing different demonstrators from each other at a low level, perhaps only to the point of discerning between same/different conspecifics. That said, this finding is more likely due to changes in the ratio of demonstrators altering the amount of exposure to each flavour. Testing whether observers would learn an equally strong STFP from multiple interactions with the same demonstrator as compared to multiple interactions with unique demonstrators would allow researchers to identify the more likely explanation.

Notably, Kuan & Colwill [30] also used an interaction paradigm with competing demonstrators that is similar to our design for Experiment 2. In their experiment, observers were given two exposures to multiple demonstrators as in the final interaction stage of our experiment, with the key differences that in Kuan and Colwill for both interactions (1) demonstrators were introduced simultaneously, (2) one demonstrator was sickened with LiCl and (3) two observers were present. This experiment produced findings indicating that observers displayed a heightened preference for the food demonstrated by the healthy demonstrator over the unhealthy demonstrator, which Galef & Whiskin [31] were unable to replicate. That this interaction paradigm has repeatedly produced difficult to interpret results does suggest that there is something unique about the way that observers process competing pheromonal inputs.

One final point of discussion is the muted effect of demonstrator order that is seen in our saline/saline control group. In considering this topic, it should be reiterated that only six subjects were run through the saline/saline condition of this experiment and no significant effect was picked up in a between group comparison of LiCl-injected observers and saline-only observers. It is quite possible that a larger sample size would bring our findings from the saline/saline observers more in line with what was observed in our LiCl-injected observers. If a valid effect, the more symmetrical reliance on information obtained from D1 and D2 in the saline/saline condition would challenge our interpretation of demonstrator order effects somewhat, but could be explained by decreased uncertainty in this group. Observers in the LiCl/saline and saline/LiCl groups had varied experiences with their demonstrated food that likely engendered a certain amount of uncertainty with regards to the safety of novel foods, an observer characteristic which is known to increase reliance on STFP [11]. It is possible that as both foods in the choice test following the final interaction were demonstrated, this increase in reliance manifested primarily for the stronger preference, i.e. the STFP acquired from the demonstrator that had more recently been interacted with. That we did not observe a heightened preference for the second demonstrated food if the demonstrator had previously been learned from in the between-subjects experiment is likely due to this lack of uncertainty and increased sensitivity of the choice test in the within-subjects experiment to small differences. Whether we would see results similar to those from our saline/saline group if observers were run through a LiCl/LiCl condition would help clarify whether uncertainty is the cause of this effect.

## 6. Conclusion

Our experimental results indicate that an aversive previous experience resulting from reliance on social information does not influence an observer's tendency to rely on information acquired from a specific demonstrator or their tendency to rely on socially acquired information in general. Unexpectedly, our results also suggest that processing of olfactory cues mediating socially transmitted food preferences may be facilitated in demonstrators that have been more recently learned from, at least in observers that have recently experienced social learning associated illness. These findings somewhat contradict previous research into the behavioural mechanisms of STFP, which have universally found observers to be insensitive to demonstrator characteristics. This may indicate that olfactory cues relating to demonstrator identity are processed at a very basic level so as to allow observers to determine the number of unique individuals they are obtaining information from in a group setting.

## Data accessibility

Our data and statistical analyses are readily available at: https://dataverse.tdl.org/dataverse/MonfilsFearMemoryLab.
